# A Phase IIa, Single-Blind, Placebo-Controlled, Parallel-Group Study to Assess Safety, Tolerability, and Pharmacokinetics/Pharmacodynamics of Brensocatib in Adults with Cystic Fibrosis

**DOI:** 10.1007/s40262-025-01550-z

**Published:** 2025-08-03

**Authors:** Michael W. Konstan, James J. Tolle, Emily DiMango, Patrick A. Flume, Helen Usansky, Ariel Teper, Christina N. Ramirez, Jimmy Flarakos, Jessica Basso, Sherry Li, Marcela Vergara

**Affiliations:** 1 Case Western Reserve University School of Medicine, Cleveland, OH USA; 2 Vanderbilt University Medical Center, Nashville, TN USA; 3 Columbia University Irving Medical Center, New York, NY USA; 4 Medical University of South Carolina, Charleston, SC USA; 5 Insmed Incorporated, Bridgewater, NJ USA

## Abstract

**Background and Objectives:**

Brensocatib, an oral, competitive, and reversible inhibitor of dipeptidyl peptidase 1 (DPP1), reduces exacerbations and lung function decline in non-cystic fibrosis bronchiectasis (NCFBE). This study aimed to evaluate the pharmacokinetics (PK), pharmacodynamics (PD), safety, and tolerability of brensocatib in adults with cystic fibrosis (CF), comparing these findings with data from previous trials in healthy adults and in those with NCFBE to inform dose selection for future clinical trials.

**Methods:**

A phase IIa, single-blind, randomized, placebo-controlled trial was conducted to assess the PK, PD, safety, and tolerability of brensocatib in adults with CF. Participants were randomly assigned to receive once-daily brensocatib (10 mg, 25 mg, or 40 mg) or placebo for 28 days. The study planned enrollment of up to 34 adults, stratified on the basis of their CF transmembrane conductance regulator (CFTR) modulator use, to evaluate the PK profile of brensocatib and its safety compared with placebo. Primary PK parameters, including maximum plasma concentration (*C*_max_), time to maximum concentration (*T*_max_), area under the concentration–time curve from 0 to 24 h (AUC_0–24_), and half-life (*t*_1/2_), were determined on day 1 and day 28. Dose-dependency of brensocatib exposure was analyzed, and safety and tolerability were assessed through treatment-emergent adverse events. Data from participants were compared with previous data from healthy adults and from those with NCFBE.

**Results:**

A total of 29 participants were randomized to treatment, with 21 stratified to the CFTR modulator group. Baseline characteristics were similar among cohorts. Mean age was 37.9 (standard deviation (SD) 14.6) years, and most participants exhibited mild-to-moderate lung disease. PK analysis showed dose-dependent and predictable brensocatib exposure, with comparable profiles between participants with and without use of CFTR modulators. In addition, PK profiles in participants were comparable to those of healthy adults and of those with NCFBE. Pharmacodynamic analysis revealed dose-dependent reduction in neutrophil serine protease (NSP) activity, reaching saturation around the 25-mg dose, particularly in blood. Brensocatib at all doses was well tolerated with no new identified safety signals.

**Conclusions:**

Brensocatib demonstrated consistent PK profiles independent of CFTR therapy and comparable to those of healthy and NCFBE adults. Brensocatib reduced blood and sputum NSP levels. The safety profile was comparable to previous studies, with no new safety concerns identified, supporting the use of similar dosing for adults with CF as for other populations. These findings advocate for further investigation of brensocatib in CF.

**Clinical Trial Registration:**

NCT05090904.

## Key Points

**Table:** 

Brensocatib is an oral, competitive, and reversible inhibitor of dipeptidyl peptidase 1, currently in development for the treatment of patients with non-cystic fibrosis bronchiectasis, chronic rhinosinusitis without nasal polyps, and hidradenitis suppurativa. This study aimed to determine the pharmacokinetics, pharmacodynamics, safety, and tolerability of brensocatib in adults with cystic fibrosis treated with or without cystic fibrosis transmembrane conductance regulator modulators.
Brensocatib systemic exposure in participants with cystic fibrosis was comparable to observations from studies of healthy adults and those with non-cystic fibrosis bronchiectasis and not affected by concomitant use of cystic fibrosis transmembrane conductance regulator modulators. Brensocatib was associated with a dose- and exposure-dependent reduction trend in all blood neutrophil serine proteases, supporting future clinical development of brensocatib for the treatment of cystic fibrosis.

## Introduction

Cystic fibrosis (CF) is caused by mutations in the gene encoding for the CF transmembrane conductance regulator (CFTR) protein. Abnormal or absent CFTR protein leads to chronic airway infection and inflammation, characterized by persistent neutrophilic influx [1, 2]. Chronic infection, primarily from *Pseudomonas aeruginosa*, is a major contributor to persistent airway inflammation in people with CF (pwCF) [3]. The morbidity and mortality from CF are due primarily to progressively destructive lung disease, which results in bronchiectasis and ultimately respiratory failure [4]. Pulmonary exacerbations, defined as the acute worsening of respiratory symptoms, such as increased cough, sputum volume, and dyspnea, further contribute to the morbidity and mortality in CF, significantly affecting lung function and quality of life [5, 6].

Current recommended treatment options for pwCF include mucolytics and inhaled antibiotics [7]. The introduction of CFTR modulator therapies has improved health outcomes for many pwCF [8]. Despite their use, pwCF may still experience pulmonary exacerbations [9] and some may not tolerate modulators owing to adverse effects [10]. Other pwCF may not be eligible for modulator use owing to their type of CFTR mutation [11]. Thus, not all pwCF fully benefit from CFTR modulators.

Even in the absence of infection, inflammation may be present [12], and inflammation persists in pwCF treated with CFTR modulators [13]. Elevated activity of neutrophil elastase (NE), a neutrophil serine protease (NSP) that is a major product of activated neutrophils, is a risk factor for the development and progression of bronchiectasis and lung function decline in pwCF and in those with non-cystic fibrosis bronchiectasis (NCFBE) [14]. In addition to NE, neutrophils secrete other NSPs, including cathepsin G (CatG) and proteinase 3 (PR3), which contribute to the pathophysiology of bronchiectasis by increasing inflammation further, leading to structural damage and promoting infection [15, 16]. Dipeptidyl peptidase (DPP1) is a lysosomal cysteine protease that activates NSPs, including NE, CatG, and PR3, during neutrophil maturation [16]. DPP1 inhibition is, therefore, a suitable target to attenuate NSP activity in CF and NCFBE.

Brensocatib is an oral, competitive, and reversible inhibitor of DPP1 [17], currently in development for the treatment of patients with NCFBE, chronic rhinosinusitis without nasal polyps, and hidradenitis suppurativa. The safety, tolerability, and pharmacokinetics (PK) of brensocatib have been investigated in multiple clinical studies for single doses of 5–120 mg and once-daily (QD) dosing at 10–40 mg [18–20]. These studies reported dose-dependent brensocatib exposure, with approximately twofold steady state accumulation and low-to-moderate interparticipant variability following single or multiple dosing. Brensocatib’s elimination half-life was 20–30 h, and at steady state, there was approximately 20% urinary excretion. Brensocatib is a substrate of CYP3A, P-glycoprotein (P-gp), and breast cancer resistance protein (BCRP), but not a clinically meaningful inhibitor or inducer of CYP3A. In addition, it has a low potential to modulate other CYP isozymes (e.g., CYP1A2, CYP2B6, CYP2C8, CYP2C9, CYP2C19, CYP2D6, and CYP2E1) and transporters (e.g., P-gp, BCRP, and solute-carrier transporters) at clinically relevant doses [18]. A strong inhibitor or inducer of CYP3A and P-gp (e.g., verapamil, clarithromycin, or rifampin) had a mild effect on brensocatib systemic exposure (maximum plasma concentration (*C*_max_) and area under the curve (AUC)), which was considered not clinically meaningful. Furthermore, a population PK analysis using pooled PK data from 12 clinical studies (phases 1, 2, and 3) showed that brensocatib PK was not affected by mild and moderate CYP3A and P-gp modulators.

In a study of adults with varying degrees of renal impairment, single-dose brensocatib was well tolerated [21]. Furthermore, brensocatib elimination and exposure were not significantly affected by renal impairment, indicating that dose adjustments are not necessary in these individuals.

In a phase III trial (ASPEN; NCT04594369), brensocatib (10 mg or 25 mg) QD significantly reduced the annualized rate of adjudicated pulmonary exacerbations compared with placebo, prolonged time to first exacerbation, and increased the proportion of participants with NCFBE remaining exacerbation-free; brensocatib 25 mg was associated with significantly lower lung function decline over 52 weeks compared with placebo as measured by forced expiratory volume in 1 s (FEV_1_) [22].

People with NCFBE and those with CF who develop bronchiectasis share many pathophysiological features, including neutrophilic inflammation, a pattern of cyclical infection, and persistent mucus obstruction of the airways [23]. Given the role of NE and other NSPs in driving the inflammation and structural damage characteristic of these conditions, brensocatib’s mechanism of inhibiting DPP1 suggests it may significantly diminish underlying neutrophilic inflammation, thus reducing the incidence of exacerbations in pwCF. In addition, alterations in drug absorption, distribution, and clearance have been observed in pwCF, including reduced oral absorption and enhanced renal clearance of certain drugs, which have led to lower serum concentrations [24–28]. Therefore, it is important to assess the PK and pharmacodynamics (PD) of brensocatib in pwCF, with or without concomitant use of CFTR modulators.

The objectives of this study were to determine the PK, PD, safety, and tolerability of brensocatib in adults with CF treated with or without CFTR modulators.

## Methods

### Study Design

This was a phase IIa, single-blind, randomized, placebo-controlled trial with a parallel-group, multiple-dose design aimed at evaluating the PK, PD, safety, and tolerability of brensocatib in adults with CF (NCT05090904).

Participants were assigned to receive a once-daily dose of brensocatib (10 mg, 25 mg, or 40 mg) or placebo for 28 days.

**Fig. 1:**
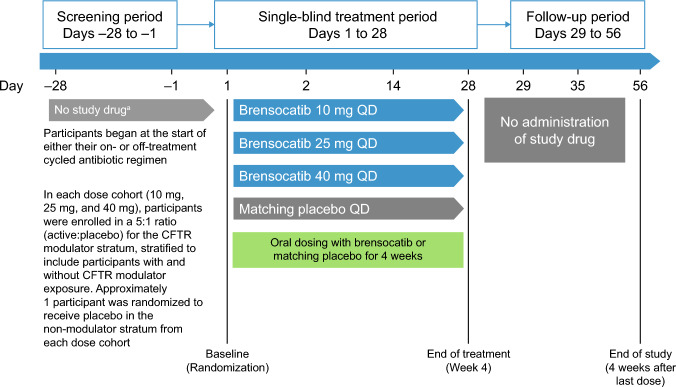
Study design. The negative days denote the screening period, while the positive days indicate the treatment and follow-up periods. These phases were contiguous, occurring in immediate succession with no intervals. ^a^Participants began study treatment at the start of either their on- or off-treatment cycled antibiotic regimen, if applicable. *CFTR* cystic fibrosis transmembrane conductance regulator, *QD* once daily

Up to 34 adults were planned to participate in the study (7 in each of the first three brensocatib treatment groups (10 mg, 25 mg, and 40 mg QD) and 4 in the placebo treatment group). All participants were classified into two distinct strata on the basis of their CFTR modulator use as concomitant medication: either having previously received and continuing to receive CFTR modulators, or not receiving CFTR modulators during the study (including never having used or not using CFTR modulators 30 days prior to screening). The distribution of participants into the CFTR modulators and non-modulators strata occurred before enrollment and was aligned with regard to the proportion of people in the CF population at large [29]. In the CFTR modulator stratum, participants were enrolled in a 5:1 ratio (active:placebo) for each dose group. In the non-modulator stratum, six participants were planned to receive active treatment (randomized to 10 mg, 25 mg, or 40 mg brensocatib), and one participant was planned to receive placebo. The study consisted of a 28-day screening period, followed by a 28-day treatment period and a 4-week safety follow-up period (Fig. 1).

### Study Objectives

The primary objectives of the study were to evaluate the PK of brensocatib in adults with CF following once-daily oral administration and to assess the safety of brensocatib versus placebo in this population over the 4-week treatment period. PK parameters were also compared in participants by CFTR modulator treatment status. The secondary objective was to evaluate the dose-dependency of brensocatib exposure.

In addition, to understand the comparability in PK, PD, safety, and tolerability of brensocatib, data from CF participants in the current study were compared with previous data from healthy adults and from those with NCFBE.

### Study Population

Key inclusion criteria included age ≥ 18 years, body mass index (BMI) ≥ 18 kg/m^2^, and a confirmed diagnosis of CF-related lung disease, with a percent predicted forced expiratory volume in 1 s (ppFEV_1_) between 40 and 90% at screening visit and baseline, and stable (unchanged with regard to dose and frequency) CF treatment for at least 30 days before screening.

Key exclusion criteria included severe or unstable CF, per investigator’s judgment; abnormal renal function test results at screening (defined as estimated glomerular filtration rate < 30 mL/min); clinically significant hepatobiliary disease (defined as alanine aminotransferase and/or aspartate aminotransferase values > 3 × upper limit of normal and total bilirubin > 2 × upper limit of normal, excluding confirmed Gilbert’s syndrome); current treatment for allergic bronchopulmonary aspergillosis or nontuberculous mycobacteria or tuberculosis; and unstable disease due to colonization with *Burkholderia cenocepacia*, *Burkholderia dolosa*, or *Mycobacterium abscessus* as judged by the investigator. The investigators were to assess whether the disease was stable on the basis of exacerbations or the need for systemic antibiotics. In addition, prohibited medication included long-term use of systemic steroids, defined as > 4 weeks, regardless of dose, for any chronic condition.

### PK and PD Sample Collection and Bio-Analyses

Plasma PK samples were collected on days 1 (at predose and 0.5, 1, 2, 4, 6, 8, and 24 h postdose), 14 (at predose and 2 h postdose), and 28 (at predose and 0.5, 1, 2, 4, 6, 8, 24, and 168 h postdose). The concentration of brensocatib in the plasma samples was analyzed using a validated liquid chromatography with tandem mass spectrometry method at an external service provider with a quantification range of 0.25–150 ng/mL. The overall intra- and inter-batch precision and accuracy were −2.7 to 2.0% and −1.8 to 0.8%, respectively. The long-term stability was established in human plasma for 346 days at −70 °C. The validation method details were published internally and stored within Insmed’s internal document repository.

Blood PD samples were collected at screening; predose on days 1, 2, 14, 28, and 29; and at the follow-up visit (day 35). The samples were analyzed at an external service provider to determine the concentrations of active NE, PR3, and CatG in white blood cell pellets using validated enzymatic methods with quantification limits of 87.9 ng/mL for NE and PR3 and 70.3 ng/mL for CatG. The fit-for-purpose methods details were published internally and stored within Insmed’s document repository.

### Statistical Analysis

A formal sample size calculation was not performed. The number of participants was based on feasibility, as is standard for such multiple ascending dose study designs, and was considered sufficient to meet study objectives.

The safety analysis set comprised all randomized participants who received at least one dose of brensocatib. The PK analysis set comprised all randomized participants who received at least one dose of brensocatib and had sufficient data to calculate at least one PK parameter. The PD analysis set comprised all randomized participants who received at least one dose of study drug with at least one predose and one postdose PD measure.

Descriptive statistics for continuous variables were calculated. Categorical variables were summarized by counts and by percentage of participants in the corresponding categories. No formal hypothesis was tested. Changes from baseline values were calculated and presented for both safety and efficacy data at each visit, including the percent change from baseline. PK parameters for brensocatib were assessed using noncompartmental analysis via Phoenix^®^ WinNonlin^®^ version 8.2 or later. SAS version 9.4 or higher was employed to prepare PK outputs.

Primary PK endpoints included maximum plasma concentration (*C*_max_), time to reach maximum serum concentration (*T*_max_), area under the concentration–time curve from 0 to 24 h (AUC_0–24_), and half-life (*t*_1⁄2_) on day 1 and on day 28. Analysis of dose dependency for brensocatib *C*_max_, AUC_0–24_, and area under the concentration–time curve from time 0 to last measurable concentration (AUC_last_) after single-dose administration and at steady state (day 28) were performed using a power law model. Dose normalization of *C*_max_, AUC_0–24_, and AUC_last_ was performed only when a linear and dose-dependent trend was observed.

If a participant experienced an intercurrent event, defined as use of antibiotics during an acute event that could influence NSP levels, the data were not included in the PD analysis. In assessing treatment-emergent adverse events (TEAEs), the “while-on-treatment” strategy was implemented, which encompassed the 28-day period following the final dose of study drug. With this strategy, data collected prior to the occurrence of the intercurrent event, that is, to discontinuation of brensocatib, were included in the analysis.

## Results

**Fig. 2:**
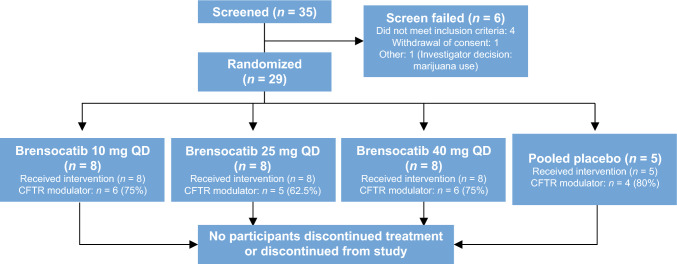
Summary of participant disposition. *CFTR* cystic fibrosis transmembrane conductance regulator, *QD* once daily

Among 35 participants screened, 29 met eligibility criteria and were randomized to treatment. Eight participants were allocated to each of the three brensocatib dose groups and five participants to the placebo group. No participants discontinued treatment or discontinued from the study (Fig. 2). All participants were stratified by CFTR use, with 21 in the CFTR modulator stratum and 8 in the no CFTR modulator stratum (Table 1).

**Table 1:** Demographics and baseline characteristics

	All participants *N* = 29	CFTR modulator stratum *n* = 21	No CFTR modulator stratum *n* = 8
Age, mean (SD), years	37.9 (14.6)	38.1 (12.2)	37.3 (20.6)
Age ≥ 30 years, *n* (%)	21 (72.4)	17 (81.0)	4 (50.0)
Male, *n* (%)	18 (62.1)	13 (61.9)	5 (62.5)
Hispanic or Latino, *n* (%)	4 (13.8)	2 (9.5)	2 (25.0)
Race, *n* (%)			
Asian	1 (3.4)	1 (4.8)	0
Black or African American	1 (3.4)	0	1 (12.5)
White	26 (89.7)	19 (90.5)	7 (87.5)
Not reported	1 (3.4)	1 (4.8)	0
BMI at screening, mean (SD), kg/m^2^	25.3 (4.7)	24.5 (4.2)	27.6 (5.6)
Antibiotics for stable CF treatment^a^, *n* (%)	17 (58.6)	13 (61.9)	4 (50.0)
Use of pancreatic enzymes, *n* (%)	22 (75.9)	17 (81.0)	5 (62.5)
ppFEV_1_^b^ at baseline, mean (SD)	64.9 (9.9)^c^	61.6 (9.0)	73.1 (7.4)
Type of CFTR^d^, *n* (%)			
Ivacaftor		1 (4.8)	
Tezacaftor/ivacaftor		1 (4.8)	
Elexacaftor/tezacaftor/ivacaftor		19 (90.5)	

Data are for the safety analysis set

*BMI* body mass index, *CF* cystic fibrosis, *CFTR* cystic fibrosis transmembrane conductance regulator, *FEV*_*1*_ forced expiratory volume in 1 s, *ppFEV*_*1*_ percent predicted FEV_1,_ *QD* once daily, SD standard deviation

^a^Consists of oral, inhaled, or nasal antibiotics

^b^FEV_1_ was pre-bronchodilator

^c^*n* = 28

^d^Only applies to CFTR modulator stratum

### Baseline Characteristics

Demographic and clinical characteristics of all participants at baseline were similar across cohorts, except for a larger proportion of male participants. Mean (SD) age was 37.9 (14.6) years, with 21 (72.4%) participants aged ≥ 30 years. A total of 18 (62.1%) participants were male, and the mean (SD) BMI was 25.3 (4.7) kg/m^2^ (Table 1).

A total of 17 (58.6%) participants were receiving a chronic antibiotics regimen for stable CF treatment, and 22 (75.9%) were receiving pancreatic enzyme replacement therapy (Table 1). Most participants had mild-to-moderate lung disease, as shown by ppFEV_1_.Those receiving CFTR modulators versus not had lower lung function (ppFEV_1_, mean [SD]: 61.6 [9.0] versus 73.1 [7.4], respectively), a higher use of pancreatic enzymes (*n* [%]: 17 [81.0] versus 5 [62.5], respectively), and higher use of chronic antibiotics (61.9% versus 50.0%, respectively) (Table 1).

### Pharmacokinetics

The PK analysis set included 24 (82.8%) participants (8 in each of the brensocatib treatment groups). Among these, 17 participants were concomitantly treated with CFTR modulators.

**Fig. 3:**
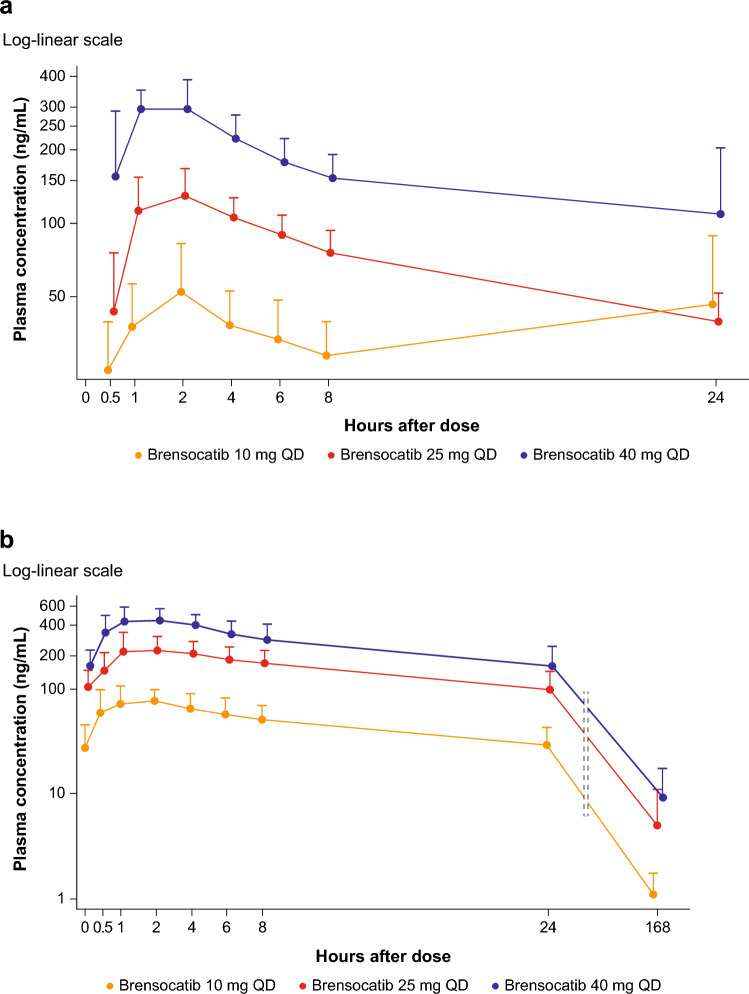
Mean (+SD) brensocatib plasma concentration profile on **a** day 1 and **b** day 28 following brensocatib once-daily oral administration with or without concomitant use of CFTR modulators. *CFTR* cystic fibrosis transmembrane conductance regulator, *QD* once daily, *SD* standard deviation

Mean plasma concentrations following a single oral administration and daily administration of brensocatib for 28 days were dose-dependent, and the inter-participant variability was low (Fig. 3a and b, respectively).

All geometric mean ratio (GMR) values for dose-normalized *C*_max_, AUC_0–24_, and AUC_last_ ranged from 1.08 to 1.21 (≤ 30%), with the 90% confidence intervals (CIs) containing 1.0, indicating that brensocatib exposure was not significantly different, regardless of concomitant use of CFTR modulators (Table 2).

**Table 2:** Dose-normalized brensocatib exposure, with/without concomitant use of CFTR modulators

	Geometric mean	*n*	*C*_max_ / D (ng/mL/mg)	AUC_0–24_ / D (h × ng/mL/mg)	AUC_last_ / D (h × ng/mL/mg)
Day 1	With CFTR modulator	17	6.7	80.7	80.7
	Without CFTR modulator	7	6.3	66.8	66.8
	Ratio with/without CFTR modulator (90% CI)		1.08 (0.83–1.40)	1.21 (0.91–1.60)	1.21 (0.91–1.60)
Day 28	With CFTR modulator	16	10.2^a^	134.2	295.1
	Without CFTR modulator	6	8.8	120.2	271.3
	Ratio with/without CFTR modulator (90% CI)		1.16 (0.87–1.54)	1.12 (0.82–1.52)	1.09 (0.73–1.63)

Data are for the pharmacokinetic analysis set

*AUC*_*0–24*_ area under the concentration–time curve from time 0 to 24 h post dose, *AUC*_*last*_ area under the concentration–time curve from time 0 to the last timepoint with measurable concentration, *CFTR* cystic fibrosis transmembrane conductance regulator, *CI* confidence interval, *C*_*max*_ maximum plasma concentration, *D* dose-normalized

^a^*n* = 17

PK analyses showed that brensocatib was rapidly absorbed. Median *T*_max_ postdose on day 1 (after the first dose) and day 28 (at steady state) was 1.5–2.0 h (Table 3).

**Table 3:** Mean (CV%)^a^ of brensocatib pharmacokinetic parameters on days 1 and 28 with/without use of CFTR modulators

Brensocatib	10 mg QD		25 mg QD		40 mg QD	
PK parameter	Day 1 *n* = 8	Day 28 *n* = 8	Day 1 *n* = 8	Day 28^b^ *n* = 7	Day 1 *n* = 8	Day 28 *n* = 8
*C*_max_ (ng/mL)	67.3 (45.4)	90.1 (31.0)	140 (24.1)	249 (40.2)	342 (17.5)	490 (30.0)
*T*_max_ (h)	2.0 (1.0–24.8)	1.9 (0.5–4.0)	1.5 (1.0–4.1)	2.0 (1.0–4.0)	1.5 (0.6–24.9)	2.0 (1.0–4.0)
AUC_0–24_ (h × ng/mL)	879 (53.1)	1100 (35.2)	1630 (21.8)	3800 (33.3)	3690 (33.0)	6320 (35.1)
AUC_last_ (h × ng/mL)	879 (53.1)	2380 (46.2)	1630 (21.8)	8930 (48.5)	3690 (33.0)	15,300 (47.7)
AUC_inf_ (h × ng/mL)	–	2430 (46.9)	–	9300 (52.3)	–	16,200 (48.8)
*C*_trough_ (ng/mL)^c^	46.3 (92.2)	27.2 (66.1)	39.3 (30.4)	104 (43.6)^d^	108 (88.6)	162 (41.8)
CL/*F* (L/h)	–	9.91 (28.6)	–	7.17 (30.1)	–	7.20 (42.8)
*V*_d_/*F* (L)	–	423 (27.1)	–	332 (28.6)	–	398 (66.0)
*t*_1/2_ (h)	–	30.0 (16.5)	–	33.1 (22.2)	–	38.5 (45.0)
*R*_ac(Cmax)_	–	1.42 (22.6)	–	1.72 (38.9)	–	1.43 (25.5)
*R*_ac(AUC0–24)_	–	1.38 (32.2)	–	2.26 (29.2)	–	1.81 (37.4)

Data are for the pharmacokinetic analysis set

*AUC*_*0–24*_ area under the concentration–time curve from time 0 to 24 h postdose, *AUC*_*inf*_ area under the concentration–time curve from time zero to infinity, *AUC*_*last*_ area under the concentration–time curve from time 0 to the last measurable concentration, *CFTR* cystic fibrosis transmembrane conductance regulator, *CL/F* apparent total clearance of drug from plasma after extravascular administration, *C*_*max*_ maximum plasma concentration, *C*_*trough*_ concentration immediately prior to dosing, *CV%* arithmetic coefficient of variation percentage, *QD* once daily, *R*_*ac(AUC0–24)*_ accumulation ratio based on AUC_0–24_, *R*_*ac(Cmax)*_ accumulation ratio based on *C*_max_, *t*_*1/2*_ elimination half-life, *T*_*max*_ time to maximum plasma concentration, *V*_*d*_*/F* apparent volume of distribution

^a^All parameters are expressed as arithmetic mean (CV%), except for *T*_max_, which is expressed as median (range)

^b^PK data from one participant were excluded for day 28 PK parameters owing to insufficient data

^c^*C*_trough_ concentrations are not available for day 1; they are presented for day 2

^d^Participant count for *C*_trough_ is *n* = 8

Across brensocatib dose groups, mean *C*_max_ on days 1 and 28 was 60.9–365 ng/mL and 92.0–538 ng/mL, respectively, in the CFTR modulator stratum, and 86.4–274 ng/mL and 84.3–348 ng/mL, respectively, in the non-CFTR modulator stratum. Mean AUC_0–24_ on days 1 and 28 was 798–4120 h × ng/mL and 1080–7360 h × ng/mL, respectively, for the CFTR modulator stratum, and 1120–2400 h × ng/mL and 1190–3740 h × ng/mL, respectively, for the non-CFTR modulator stratum. Mean AUC_last_ on days 1 and 28 was 798–4120 h × ng/mL and 2440–17,700 h × ng/mL, respectively, for the CFTR modulator stratum, and 1120–2400 h × ng/mL and 2210–9540 h × ng/mL, respectively, for the non-CFTR modulator stratum. The GMRs of dose-normalized *C*_max_ and AUC (AUC_0–24_ and AUC_last_) on days 1 and 28 were 1.08 and 1.16 for *C*_max_ and 1.21 and 1.09 for AUC (Table 2). The elimination *t*_1/2_ was comparable between participants in the CFTR modulator stratum and the non-CFTR modulator stratum, ranging from 24.7 to 49.5 h. These data indicate that brensocatib PK in adults with CF is not affected by the concomitant use of CFTR modulators. On the basis of the PK comparability between the CFTR strata, combined PK parameters (CFTR and non-CFTR modulator groups, presented in Table 3) were used as the primary PK outcomes for the data presentation, discussion, and conclusions.

Brensocatib systemic exposure was dose-dependent. The least squares geometric mean ratio (LSGMR) in *C*_max_ (90% CI) comparing the 40-mg dose to the lower doses on day 28 was 1.36 (1.02–1.82) for 10 mg and 1.25 (0.92–1.69) for 25 mg. The LSGMR in AUC_0–24_ (90% CI) comparing the 40-mg dose to the lower doses on day 28 was 1.42 (1.04–1.92) for 10 mg and 1.02 (0.74–1.41) for 25 mg.

Exposure to brensocatib showed moderate accumulation at steady state at 10, 25, and 40 mg (1.5- to 2-fold accumulation on *C*_max_ and AUC; Table 3).

The disposition parameters, such as CL/*F* (apparent total clearance of drug from plasma after extravascular administration), *V*_d_/*F* (apparent volume of distribution), and elimination *t*_1/2_ were consistent over the dose range. On day 28, the *t*_1/2_ of brensocatib progressively increased with each higher dose administered across the treatment groups (Table 3).

The inter-participant variability (coefficient of variation expressed as a percentage) in *C*_max_, AUC_0–24_, and *t*_1/2_ was low to moderate, generally within 15–50%. Overall, brensocatib PK in participants was linear and predictable based on dose-dependent exposure and consistent disposition parameters across doses and low-to-moderate interindividual variability.

**Fig. 4:**
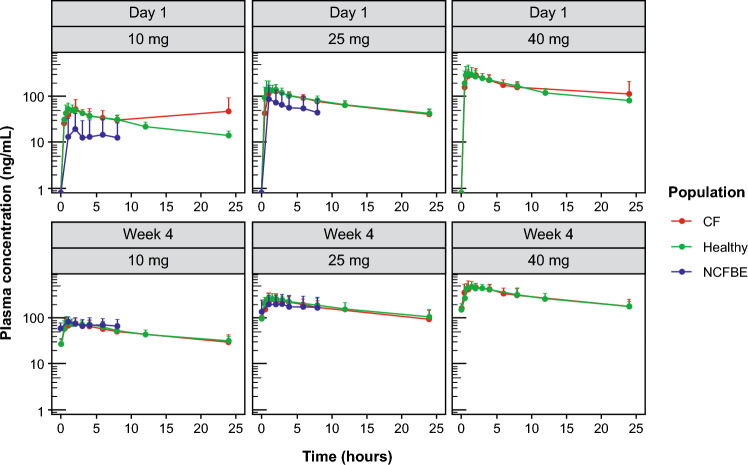
Plasma concentration of brensocatib once daily at day 1 compared with day 28 in participants with CF compared with healthy adults^a^ and those with NCFBE^b,c^. ^a^Data were included from healthy adults from a phase I study of the safety, tolerability, and PK of brensocatib 10, 25, and 40 mg [18]. ^b^Data were included from adults with NCFBE treated with brensocatib 10 mg or 25 mg in the phase II WILLOW study [15]. ^c^Data for the 40-mg arm were not available for adults with NCFBE. *CF* cystic fibrosis, *NCFBE* non-cystic fibrosis bronchiectasis, *PK* pharmacokinetics

The plasma concentration profiles in adults with CF from this study were compared with those in healthy adults following once-daily dosing at 10, 25, and 40 mg [18] and in those with NCFBE following once-daily dosing at 10 and 25 mg [15]. Plasma concentration–time profiles of brensocatib (10 mg, 25 mg, or 40 mg) in the 24 h postdose on day 1 (after a single dose) and on day 28 (at steady state, after 4 weeks of daily dosing) in participants with CF compared with healthy adults and those with NCFBE are shown in Fig. 4. The superimposable PK concentration profiles, especially on day 28, indicate that brensocatib PK in adults with CF is highly comparable to that in non-CF populations.

### Pharmacodynamics

**Fig. 5:**
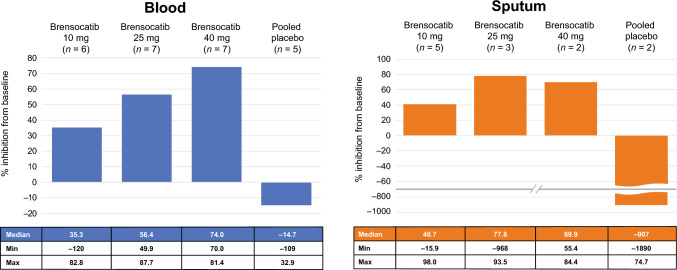
Median % reduction of NE activity in blood and sputum relative to baseline on day 29. Data are for the pharmacodynamic analysis set. Values below the limit of quantitation are included in the analysis using lower limit of quantification / 2. Percent reduction is defined as $100 \times \left(1 - \left(\frac{post - baseline\,value}{baseline\,value}\right)\right)$. *NE* neutrophil elastase

On the basis of the comparability of PK between participants with and without concomitant use of CFTR modulators, NSP activity (NE, CatG, PR3) in blood and sputum was analyzed using the combined data sets without the CFTR modulator stratum. Analysis of NE activity in blood demonstrated that, compared with placebo, there was a dose-dependent increase in the median percent reduction of NE activity with brensocatib, from 35.3% to 74.0% over the dose range of 10–40 mg QD. For NE activity in sputum, a similar trend was seen for median percent reduction, ranging from 40.7% to 77.8% across brensocatib doses, with the greatest reduction seen with the 25-mg dose (Fig. 5).

Similar findings were also reported from studies in healthy adults and in adults with NCFBE (Table 4) [15, 30]. Healthy adults exhibited a median percent reduction of NE activity ranging from 34.8 to 55.1% across brensocatib doses. In adults with NCFBE, the median percent reduction in NE activity was 30.9% and 66.5% in the brensocatib 10 mg and 25 mg groups, respectively (Table 4).

**Table 4:** Median % reduction of NE activity in the blood of healthy adults^a^ and in adults with CF or NCFBE^b,c^

Dose	Healthy adults	CF	NCFBE
Brensocatib 10 mg	34.8 (*n* = 6)	35.3 (*n* = 6)	30.9 (*n* = 38)
Brensocatib 25 mg	42.6 (*n* = 7)	56.4 (*n* = 7)	66.5 (*n* = 45)
Brensocatib 40 mg	55.1 (*n* = 7)	74.0 (*n* = 7)	N/A
Placebo	16.5 (*n* = 8)	−14.7 (*n* = 5)	3.4 (*n* = 40)

Data are for the pharmacodynamic analysis set

*CF* cystic fibrosis; *N/A* not available, *NCFBE* non-cystic fibrosis bronchiectasis, *NE* neutrophil elastase

^a^Data were included from healthy adults from a phase I study of the safety, tolerability, and PK of brensocatib 10, 25, and 40 mg [30]

^b^Data were included from adults with NCFBE treated with brensocatib 10 mg or 25 mg in the phase II WILLOW study [15]

^c^Data for the 40-mg arm were not available for adults with NCFBE

The median percent reduction of CatG activity in blood displayed a consistent trend with increasing doses of brensocatib, especially in the 25 mg and 40 mg groups, and ranged from 75.1% to 91.3% in all doses of brensocatib (Table 5). For PR3, the median percent reduction of activity in blood with brensocatib ranged from 17.3% to 55.0% over the dose range of 10–40 mg QD. Despite the absence of a dose-related effect on sputum CatG and PR3 activities, reductions were observed: CatG activity decreased in the 10-mg dose group, and PR3 activity decreased in both the 10-mg and 25-mg dose groups (Table 5).

**Table 5:** Median % reduction of NSP activity in blood and sputum relative to baseline on day 29

Matrix	Dose	NSP, median % reduction		
		NE	CatG	PR3
Blood	10 mg	35.3 (*n* = 6)	75.1 (*n* = 6)	17.3 (*n* = 6)
	25 mg	56.4 (*n* = 7)	84.9 (*n* = 7)	40.8 (*n* = 7)
	40 mg	74.0 (*n* = 7)	91.3 (*n* = 7)	55.0 (*n* = 7)
	Placebo	−14.7 (*n* = 5)	11.3 (*n* = 5)	2.52 (*n* = 5)
Sputum	10 mg	40.7 (*n* = 5)	51.3 (*n* = 5)	59.1 (*n* = 5)
	25 mg	77.8 (*n* = 3)	0 (*n* = 3)	67.1 (*n* = 3)
	40 mg	69.9 (*n* = 2)	0 (*n* = 2)	−17.0 (*n* = 1)
	Placebo	−907 (*n* = 2)	21.2 (*n* = 2)	28.7 (*n* = 2)

Data are for the pharmacodynamic analysis set

Values below the limit of quantitation are included in the analysis using lower limit of quantification / 2

Percent reduction is defined as $100 \times \left(1 - \left(\frac{post - baseline\,value}{baseline\,value}\right)\right)$

*CatG* cathepsin G, *NE* neutrophil elastase, *NSP* neutrophil serine proteinase, *PR3* proteinase 3

**Fig. 6:**
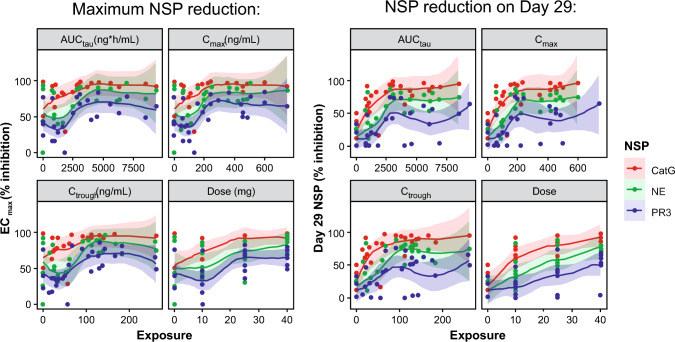
Reduction of NSP activity with increasing brensocatib exposure. *AUC*_*tau*_ area under the plasma concentration–time curve during a dosage interval, *CatG* cathepsin G, *C*_*max*_ maximum plasma concentration, *C*_*trough*_ concentration immediately prior to dosing, *EC*_*max*_ maximum inhibition, *NE* neutrophil elastase, *NSP* neutrophil serine proteinase, *PR3* proteinase 3

The relationships between brensocatib systemic exposure (area under the plasma concentration–time curve from 0 to 24 h (AUC_0–24_) during a dosage interval (AUC_tau_), *C*_max_, and concentration immediately prior to dosing (*C*_trough_)) and the maximum reduction of NSP activity (EC_max_, % inhibition) or the NSP reduction on day 29 (% inhibition) were explored using locally estimated scatterplot smoothing (LOESS) regression (Fig. 6). All three NSP enzymes showed exposure- and dose-dependent reduction. The NSP activity reduction reached a saturation point at approximately 25 mg, as indicated by the plateau of the associated PK measures. The sensitivity of reduction for brensocatib was greatest for CatG, followed by NE, and PR3 (Fig. 6).

### Safety

The incidence of TEAEs is presented in Table 6. Reported TEAEs were mostly mild to moderate in intensity. Only one participant experienced a serious TEAE, in the brensocatib 40-mg treatment group, which was an infective pulmonary exacerbation of CF. No new safety signals were identified, and there were no reported discontinuations due to adverse events, adverse events of special interest, or deaths (Table 6).

**Table 6:** Safety summary of treatment-emergent adverse events

*n* (%)	Brensocatib			Pooled placebo *n* = 5	Total *N* = 29
	10 mg QD *n* = 8	25 mg QD *n* = 8	40 mg QD *n* = 8		
Any TEAE	4 (50.0)	5 (62.5)	4 (50.0)	2 (40.0)	15 (51.7)
TEAE related to study treatment	0	1 (12.5)	1 (12.5)	1 (20.0)	3 (10.3)
Serious TEAE	0	0	1 (12.5)	0	1 (3.4)
TEAE related to COVID-19	0	1 (12.5)	1 (12.5)	0	2 (6.9)
Most common TEAEs (≥ 5%)					
Infective pulmonary exacerbation of CF	2 (25.0)	0	1 (12.5)	0	3 (10.3)
Headache	0	2 (25.0)	0	1 (20.0)	3 (10.3)
COVID-19	0	1 (12.5)	1 (12.5)	0	2 (6.9)
Abdominal pain	0	0	1 (12.5)	1 (20.0)	2 (6.9)
Cough	1 (12.5)	1 (12.5)	0	0	2 (6.9)
Sputum increased	2 (25.0)	0	0	0	2 (6.9)

Data are for the safety analysis set

*CF* cystic fibrosis, *COVID-19* coronavirus disease 2019, *QD* daily, *TEAE* treatment-emergent adverse event

## Discussion

The PK, PD, and safety of brensocatib once daily at 10, 25, and 40 mg were evaluated in adults with CF, with PK evaluation as a primary endpoint. The PK data from this study were used to evaluate the impact of CFTR modulators on brensocatib PK and to evaluate the PK differences between CF and non-CF populations. In addition, the PD and safety differences between adults with CF and NCFBE and exploratory PK/PD relationships were evaluated.

The results of this phase II study in adults with CF (with or without concomitant use of CFTR modulators) showed that brensocatib was characterized by a rapid absorption, dose-dependent exposure, moderate rate of elimination, low-to-moderate inter-participant variability, and moderate exposure accumulation at steady state. No differences in PK parameters were observed between participants with or without use of concomitant CFTR modulators. Furthermore, based on the concentration profiles at days 1 and 28, brensocatib exposure in adults with CF was comparable to that of healthy adults and those with NCFBE [19, 21, 22]. The PK results indicate that the systemic exposure of brensocatib is not altered by highly effective CFTR modulators or by the disease status of CF. The safety profile for brensocatib in this CF population over 28 days was comparable to that observed in previous trials, including the phase II WILLOW study and the phase III ASPEN study in adults with NCFBE [15, 22], with no new safety signals.

Brensocatib was associated with a dose- and exposure-dependent reduction trend in blood and sputum NSP activity in adults with CF, despite high variability in the NSP data. Among the evaluated NSPs, the greatest reduction in activity from baseline in sputum was observed with NE, ranging from 41 to 78% across all brensocatib doses, with the greatest reduction seen in the 25-mg dose. CatG demonstrated the most pronounced reduction in activity from baseline in blood across all doses of brensocatib (75–91%), followed by NE (35–74%), and PR3 (17–55%). These results were consistent with previous findings in healthy adults and in those with NCFBE [19].

In this study, the doses administered to the adults with CF were similar to the doses used to treat adults with NCFBE. The administered doses in this study were sufficient based on the Safety Review Committee’s examination of the safety and PK data of the doses used; hence, dose escalation to 65 mg was not implemented. Additional studies would be needed to confirm the efficacy of brensocatib in adults with CF and bronchiectasis.

### Limitations

The sample size for non-modulator participants in this study was relatively small owing to the availability in this group during the enrollment. To overcome this, the PK comparison between the modulator strata was conducted using pooled *C*_max_ and AUC across the dose levels (i.e., *C*_max_/D and AUC/D, *n* = 7 for the group without CFTR modulator and *n* = 17 for the group with modulator). Since brensocatib PK is linear and predictable at the dose levels with low-to-moderate intersubject variability, the conclusion for the PK comparability between the modulator strata is considered valid.

The interpretation of exploratory NSP data was limited by the small size of the data set, high variability at baseline for both blood and sputum, and assay variabilities. Participants treated with CFTR modulators had reduced sputum production as reported in literature [31] and observed in this study. The small sample size of the sputum NSP data made the comparisons between the CFTR strata challenging. However, based on the comparability of PK and blood NSP data, the NSP levels in sputum are likely to be similar in adults with or without CFTR modulators. The largest proportion of participants was receiving elexacaftor/tezacaftor/ivacaftor and none were receiving lumacaftor/ivacaftor; therefore, the impact of individual CFTR modulators requires additional study.

Despite their variability, both blood and sputum NSP data were generated using robust methods. Although many participants were unable to provide sputum samples, the data collection methods used were qualified. Trends observed in the blood and sputum NSP data aligned with findings from other clinical studies, reinforcing the current study’s findings despite the limitations.

## Conclusions

Brensocatib PK in adults with CF participating in this study were linear and predictable, characterized by a rapid oral absorption, dose-dependent systemic exposure, moderate rate of elimination, and low-to-moderate inter-individual variability. Brensocatib PK were not affected by concomitant use of CFTR modulators. Furthermore, brensocatib systemic exposure was comparable to that observed in other studies of healthy adults and those with NCFBE.

Brensocatib was also associated with a dose- and exposure-dependent reduction trend in all blood NSPs (NE, CatG, and PR3) and sputum NE activity, corroborating previous findings in adults with NCFBE and in healthy adults.

Brensocatib was generally well tolerated across the different doses, with no new safety signals reported. Considering the unaffected PK of brensocatib by CFTR modulators and the current safety findings, this supports the prospect of dosing adults with CF similarly to other populations. The PK, PD, and safety data from this study support future clinical development of brensocatib for the treatment of CF.

## Declarations

### Availability of Data and Material

The datasets generated and/or analyzed during the current study are not publicly available owing to patient privacy.
